# Opposed circulating plasma levels of endothelin-1 and C-type natriuretic peptide in children with *Plasmodium falciparum *malaria

**DOI:** 10.1186/1475-2875-7-253

**Published:** 2008-12-15

**Authors:** Anelia Dietmann, Peter Lackner, Raimund Helbok, Katharina Spora, Saadou Issifou, Bertrand Lell, Markus Reindl, Peter G Kremsner, Erich Schmutzhard

**Affiliations:** 1 Clinical Department of Neurology, Innsbruck Medical University, Innsbruck, Austria; 2 Department of Parasitology, Institute of Tropical Medicine, University of Tübingen, Medical School Tübingen, Tübingen, Germany; 3 Medical Research Unit, Albert Schweitzer Hospital, Lambaréné, Gabon

## Abstract

**Background:**

Molecular mechanisms involved in the pathogenesis of severe *Plasmodium falciparum *malaria (SM), are not yet fully understood. Both endothelin-1 (ET-1) and C-type natriuretic peptide (CNP) are produced by vascular endothelium and act locally as paracrine regulators of vascular tone, ET-1 being a potent vasoconstrictor and CNP having strong vasorelaxant properties.

**Methods:**

Plasma levels of ET-1 and N-terminal fragments of CNP (NT-proCNP) were studied on admission and after 24 hours of treatment, using enzyme-linked-immunosorbent-assay (ELISA) technique, in Gabonese children with severe falciparum malaria (SM, *n *= 50), with uncomplicated malaria (UM, *n *= 39) and healthy controls (HC, *n *= 25).

**Results:**

Compared to HC, malaria patients had significantly higher plasma levels of ET-1 and significantly lower levels of NT-proCNP (*p *< 0.001 and *p *< 0.024 respectively). Plasma levels of NT-proCNP were additionally decreased in SM patients compared to HC (*p *= 0.034), whereas UM was not significantly different to HC. In the SM group we found a trend towards lower ET-1 levels compared to UM (*p *= 0.085).

**Conclusion:**

In the present study, an imbalance between the vasoconstricitve and vasorelaxant endothelium-derived substances ET-1 and CNP in the plasma of children with falciparum malaria is demonstrated, presumably in favor of vasoconstrictive and pro-inflammatory effects. These results may indicate involvement of ET-1 and CNP in malaria pathogenesis. Furthermore, results of lower ET-1 and CNP levels in SM may reflect endothelial cell damage.

## Background

Malaria, beside HIV and tuberculosis, is still a major cause of death in developing countries, accounting for 300 – 500 million clinical cases and more than one million deaths per year [1]. *Plasmodium falciparum *may lead to severe malaria (SM), a complex clinical syndrome with yet incompletely understood pathomechanisms.

It has been shown that the endothelium is critically involved in the development of severe malaria [2-4]. The endothelial response to injury in acute inflammation can be divided into two phases: an initial rapid endothelial cell activation with recruitment of neutrophils, changes in levels of nitric oxide (NO), endothelin-1 (ET-1) and C-type natriuretic peptide (CNP) amongst others, and a slower response that depends on new gene expression and fundamental changes in cell surface characteristics [5]. Endothelins are regulators of vascular tone, amplifiers of inflammatory response and are involved in normal cell proliferation as well as repair and tissue development [6]. Endothelin-1 (ET-1) is a 21-amino acid vasoconstrictive peptide [6]. It is synthesized by vascular endothelial cells as well as neurons, astrocytes and other cells at low basal levels and ET-1 tissue expression is up-regulated in response to a variety of stress stimuli [6]. ET-1 can modulate the endothelial expression of adhesion molecules and cytokines production [7,8]. Moreover, ET-1 has been shown to be up-regulated in a variety of infectious diseases, such as sepsis [9,10], Chagas' disease [11] or community acquired pneumonia [12] In contrast, clinical data on ET-1 in malaria is limited. It has been shown that big ET-1, a 38-amino acid precursor of ET-1, is elevated in patients with falciparum malaria [13]. Further, experimental in vivo data indicate that C57Bl/6 mice infected with *Plasmodium berghei *ANKA showed markedly increased ET-1 mRNA expression [14].

However, co-culture of microvascular endothelial cells treated with *P. falciparum *parasitized red blood cells (pRBC), but not with uninfected red blood cells (RBC), induced a dose-dependent decrease of ET-1 production [15]. Interactions of endothelins and natriuretic peptides are well described in the literature. CNP is a 22-amino acid peptide that is likely to act as an autocrine or paracrine agent in the CNP-synthesizing tissues, mainly endothelial cells [16]. Unlike the other two members of the Natriuretic Peptide family, the atrial natriuretic peptide (ANP) and the brain natriuretic peptide (BNP), CNP does not have direct natriuretic activity. CNP has been found to be a novel endothelium-derived hyperpolarizing factor that complements the actions of other endothelial vasorelaxant mediators such as NO and prostacyclin [17]. Scotland *et al *demonstrated that CNP furthermore inhibits leukocyte recruitment and platelet-leukocyte interactions via suppression of P-selectin expression in human umbilical vein endothelial cells [18]. CNP has not been investigated in malaria so far and the role of ET-1 in human falciparum malaria remains unclear. The current study investigates the levels of ET-1 and CNP in the plasma of children with severe and uncomplicated malaria in comparison to healthy controls.

## Methods

### Study site and participants

All children were consecutively recruited in the paediatric ward of either the Albert Schweitzer Hospital or the Regional Hospital of Lambaréné/Gabon between October 2005 and May 2006. The area is hyperendemic for *P. falciparum *with transmission year-round [19,20]. The 114 children enrolled in the study were both sexes and aged between eight months and seven years. 89 malaria cases and 25 healthy controls (see Table 1) were included. All groups were sex and age matched. All patients and control subjects were included in the study only after signed, informed, parental consent was obtained. The study was approved by the ethics committee of the International Foundation of the Albert Schweitzer Hospital in Lambaréné, Gabon.

**Table 1:** Demographic, clinical, and laboratory characteristics of study groups

Characteristics	Study groups			
	SM	UM	*p *Value	HC
	
Number of subjects	50	39		25
Gender female/male	20/30	23/16	NS^×^	14/11
^+^Age (months)	30 (9–86)	35 (9–83.5)	NS^‡^	31.5 (10–77)
^+^Onset of signs and symptoms in days before admission	3 (0–30)	3 (0–21)	NS	
^+^sMODS	18 (13–26)	13 (10–20)	< 0.001	
^+^Parasite count (μL^-1^)	69,225 (155–900,000)	23,910 (425–180,000)	< 0.05	
WBC × 10^3 ^(μL^-1^)	10.5 ± 5.9	7.7 ± 3.1	< 0.05	8.9 ± 2.6
RBC × 10^6 ^(μl^-1^)	2.9 ± 1.1	3.8 ± 0.6	< 0.001	4.7 ± 0.5
Mean Hb (g/dL)	6.8 ± 2.5	8.7 ± 1.5	< 0.001	10.7 ± 1.0
Platelets × 10^3 ^(μl^-1^)	159 ± 109	220 ± 132	< 0.005	381 ± 98

ET-1 on admission (fmol/ml)	0.76 ± 0.48	1.11 ± 0.98	<0.001^‡^	0.49 ± 0.31

ET-1 after 24 hours (fmol/ml)	0.72 ± 0.49	0.95 ± 0.71	NS^‡^	

CNP on admission (pmol/l)	11.05 ± 6.58	11.49 ± 4.55	<0.05^‡^	14.29 ± 6.4

CNP after 24 hours (pmol/l)	11.5 ± 6.83	9.92 ± 3.64	NS^‡^	

Data are mean (standard deviation), unless otherwise indicated. +Median (interquartile range). Wilcoxon rank sum test was used for testing of statistical significance unless not otherwise indicated. ^×^Chi-Square test; ‡ANOVA. NS: not significant. SM: severe malaria; UM: uncomplicated malaria; HC healthy control; sMODS: simplified Multi-Organ-Dysfunction-Score; RBC: red blood cells; Hb: haemoglobin; WBC: white blood cells.

### Clinical definition of malaria and study groups

Malaria was defined as a positive blood smear for *P. falciparum *and presence of fever (axillary temperature ≥ 37.5°C or rectal temperature ≥ 38°C) or previous episodes of fever before admission (assessed by parents or guardian) in a child presenting with signs and symptoms of severe or uncomplicated malaria [21]. Severe malaria (SM) was defined according to the WHO criteria (2000 [21]), including hyperparasitaemia (>10% parasitized red blood cells), severe anaemia (haemoglobin <5 g/dL), prostration (inability to sit or in smaller children to drink unaided), jaundice (clinically assessed by examination of conjunctiva and palm of hand or if assessable bilirubin > 3 mg%) or respiratory distress (RD, presence of abnormal deep breathing with nasal flaring, and chest recession). Children with ≥ one convulsion within 24 hours before admission and a Blantyre Coma Score (BCS) of ≤ 2 on admission and no other apparent cause of coma were classified as cerebral malaria. Age-matched children without signs and symptoms of infections at time of investigation and a negative peripheral blood smear for malarial parasites served as healthy controls (HC).

### Blood collection and physical examination

Venous blood samples were collected, on admission before starting treatment and 24 hours after admission, using sterile Sarstaedt S-Monovette citrate plasma tubes containing 0.106 molar citrate. Plasma was obtained by centrifugation at 3,000 rcf for 10 min within 30 min of blood collection and it was stored at -80°C until use. The physical examination on admission was conducted by the same investigator and the clinical course of patients was reported until discharge. In the course of the physical examination on admission the simplified Multi-Organ-Dysfunction-Score (sMODS) was performed [22,23], assessing clinical parameters of disease severity in 10 different organ systems.

### Management of patients

According to local guidelines, patients with SM were treated with intravenous quinine or sulphadoxine-pyrimethamine in case of uncomplicated malaria.

### Laboratory analysis

White and red blood cell counts, haemoglobin (Hb) levels and platelets were determined using an automated haematology analyzer. Parasitological analysis and assessment of parasitaemia was done according to the Lambaréné method [24]. Thick blood films were Giemsa stained and examined by two different experienced microscopists. Slides were considered to be negative if parasites were not detected after examination of 100 oil-immersion fields of the thick smear.

### ET-1 and NT-proCNP assays

Plasma concentrations of ET-1 and NT-proCNP were measured using commercial enzyme-linked immunosorbent assays (ELISA) (BI-20052 and BI-20872, Biomedica Group, Vienna, Austria) in accordance with the manufacturer's instructions. Natriuretic peptides are produced as propeptides, which are subsequentially cleaved to the biologically active, C-terminal hormone and the N-terminal fragment (NT-proCNP). The N-terminal fragments are more stable, they are easier and more reliable to be measured in plasma, therefore we decided to use the sandwich immunoassay for NT-proCNP.

### Statistical analysis

In order to reach equal variance and normal distribution, data of ET-1 and CNP plasma levels were logarithmically transformed. Levels of ET-1 and CNP were compared between the groups of interest using one-way ANOVA. P-values for post-hoc analysis were Bonferroni corrected. Additionally, non-transformed ET-1 and CNP plasma levels were tested by non-parametrical tests – Kruskal-Wallis Test and Dunn's post test for multiple comparisons between groups of interest and Wilcoxon rank sum test when only two groups were compared. As no difference in significances was found between parametrical and non-parametrical tests, results are shown for parametrical testing. Repeated measures of ET-1 and CNP levels were analysed by paired Students-t test. For non-normally distributed data Kruskal-Wallis Test and Wilcoxon rank sum test (when only two groups were compared), were used respectively. Proportions were analysed by Chi-square test. Correlations between different variables were analysed by Pearson correlation and Spearman's rank respectively depending on the distribution of the data. Statistical significance was defined as two-sided *p*-value < 0.05. Calculations were done using SPSS 15 (Insightful Corporation, Chicago, USA), graphs were drawn by GraphPad Prism version 5.00 (GraphPad Software, San Diego, USA).

## Results

### Characteristics of study population

Eighty-nine children with *P. falciparum *malaria were enrolled in the study, which had SM (*n *= 50) or UM (*n *= 39). Twenty-five children served as HC (*n *= 25). Demographic, clinical, and laboratory characteristics of the study groups are summarized in Table 1. Out of 50 patients with SM, 20 had severe anaemia (40%), 26 patients (52%) had one or more convulsions within the last 24 hours before admission and six patients (12%) showed hyperparasitaemia. Thirty-two children (64%) were prostrated, 20 had impaired consciousness (40%), 25 showed jaundice (50%) and 23 had respiratory distress (46%). Seven children (14%) fulfilled WHO criteria to be classified as CM. The sMODS was significantly higher in SM than in UM (*p *< 0.001).

### Plasma levels of ET-1 and NT-proCNP

**Figure 1:**
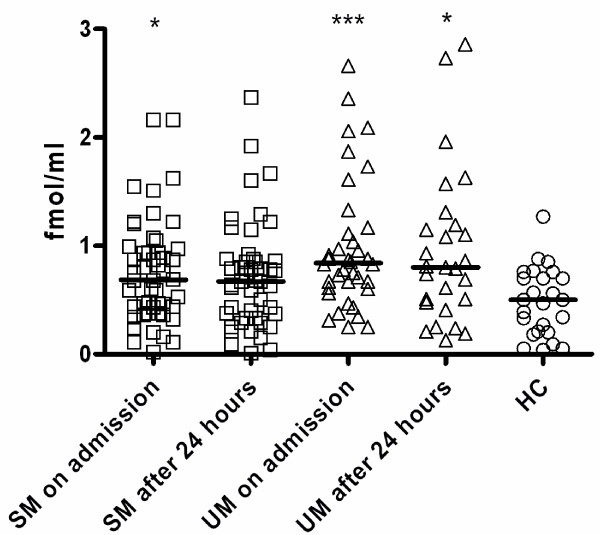
**Endothelin-1 plasma levels**. Circulating plasma levels of endothelin-1 (ET-1) in children with severe malaria (SM, square dots), uncomplicated malaria (UM, triangle dots) on admission to hospital and after 24 hours of treatment and healthy controls (HC, round dots), determined using ELISA-technique. Scatter plots, lines indicate Median values. Analyses by ANOVA, p-values were Bonferroni corrected for multiple comparisons. *p < 0.05, **p < 0.01, ***p < 0.001

ET-1 serum levels were significantly higher in all malaria patients compared with HC on admission (*p *< 0.001) and after 24 hours of treatment (*p *= 0.018). On admission UM patients showed significantly higher levels when compared to HC (*p *< 0.001) and a trend towards higher levels (*p *= 0.085) than SM patients. SM patients also had significantly higher ET-1 levels than HC (*p *= 0.029). After 24 hours of treatment, UM patients still showed significantly higher ET-1 levels when compared with HC (*p *= 0.019), but a significant difference was seen neither between SM and HC (*p *= 0.249) nor SM and UM (*p *= 0.474) patients (Figure 1).

**Figure 2:**
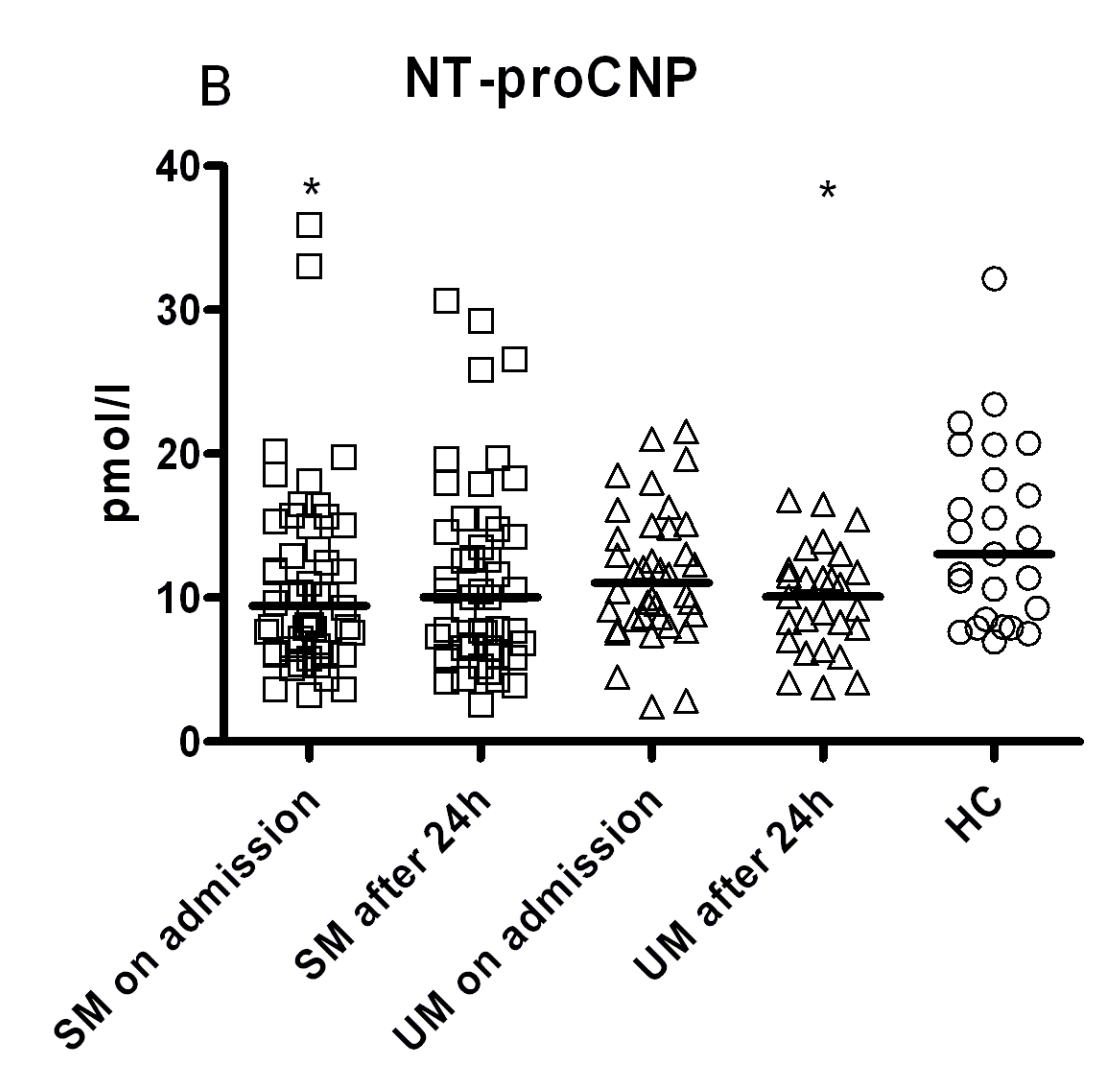
**NT-proCNP plasma levels**. Circulating plasma levels NT-pro C-type natriuretic peptide (CNP) in children with severe malaria (SM, square dots), uncomplicated malaria (UM, triangle dots) on admission to hospital and after 24 hours of treatment and healthy controls (HC, round dots), determined using ELISA-technique. Scatter plots, lines indicate Median values. Analyses by ANOVA, p-values were Bonferroni corrected for multiple comparisons. *p < 0.05, **p < 0.01, ***p < 0.001

On admission, all malaria patients showed lower CNP levels compared to HC (*p *= 0.024). SM patients had significantly lower NT-proCNP levels than HC (*p *= 0.034), but levels of UM patients were not statistically different to those from HC (*p *= 0.254). After 24 hours of treatment, all malaria patients showed lower levels compared to HC (*p *= 0.009). NT-proCNP plasma levels of UM patients were significantly lower than HC (*p *= 0.043) and SM still showed a trend towards lower NT-proCNP levels compared to HC (*p *= 0.069). Neither on admission nor after 24 hours of treatment was any significant difference seen between UM and SM (Figure 2).

### Clinical correlations with ET-1 plasma levels

Children with severe anaemia within the SM group yielded significantly lower ET-1 levels on admission (*p *= 0.041).

## Discussion

In this study, it is shown that ET-1 is significantly higher and CNP significantly lower in *P. falciparum *malaria compared to healthy controls, i.e. opposed plasma levels of ET-1 and CNP.

A key feature of *P. falciparum *malaria pathogenesis is the activation of the vascular endothelium due to – amongst others – the production of pro-inflammatory mediators such as cytokines and chemokines, resulting in up-regulation of adhesion molecules and sequestration of pRBCs, mononuclear leukocytes and platelets [25,26]. Both ET-1 and CNP are produced mainly by vascular endothelium and act locally as paracrine regulators of vascular tone [6,27]. Pathways of CNP and ET-1 synthesis from pro-CNP and pro-ET-1, respectively, are similar as they both require furin-mediated processing of precursors [28,29]. However, ET-1 is a potent vasoconstrictor [6] whereas CNP has strong vasorelaxant properties [17].

These findings correspond well with elevated plasma levels of big ET-1 found in patients with complicated malaria by Wenisch *et al *[13]. Furthermore, it has been shown that expression of several components of the endothelin pathway including ET-1, endothelin converting enzyme (ECE), and the endothelin receptors A and B (ETA and ETB) are markedly increased in mice with cerebral malaria [14]. Basilico *et al *investigated the production of ET-1 by human microvascular endothelial cells (HMEC) and human umbilical vein endothelial cells (HUVEC), co-cultured with pRBCs. ET-1 production in both HMEC-1 and HUVEC was increased by hypoxia and Interleukin 1 beta (IL1-beta). However, both normoxic ET-1 production as well as hypoxia- or IL1-beta-induced ET-1 production was reduced in a dose-dependent manner by co-incubation with pRBCs, but not with uninfected RBCs [30,31].

The results of this study demonstrate a trend towards lower ET-1 levels in SM compared to UM. As circulating pRBCs do not reflect the degree of endothelial cell activation and pRBC sequestration in the microvasculature, the decreasing ET-1 levels in SM patients could indirectly represent inhibition of endothelial ET-1 production by sequestered pRBCs, as demonstrated in vitro by Basilico *et al *[15]. This might be the reason for the lack of correlation between parasitaemia, ET-1 levels and clinical disease severity. Another reason for the observed trend towards lower ET-1 levels together with significantly lower CNP levels in SM patients could be endothelial cell damage during SM. Pro-inflammatory up-regulation of adhesion molecules with intense sequestration could provoke subsequent cell damage with either inhibition or inability to increase the production of endothelial substances such as ET-1 or CNP in SM. Endothelial cell damage and its impact on the clinical course of malaria is not yet clear. However, in vitro studies as well as murine experiments indicate important vulnerability of endothelial cells during severe/cerebral malaria [31,32].

The current findings demonstrate an imbalance between vasoconstricitve and vasorelaxant endothelium-derived substances, circulating in children with falciparum malaria. The preponderance of vasoconstrictive effects may not only be due to elevated ET-1 and decreased CNP levels, but also indirectly via impaired endothelial nitric oxide (NO) production, one of the main endothelium-derived relaxing factors. Razmy *et al *were able to establish a link between ET-1 and NO production, which was decreased in human saphenous vein endothelial cells after exposure to ET-1 [33]. NO has host protective and anti-inflammatory roles (reviewed in [34]) and its functions have been studied extensively in malaria (reviewed in [2,33-37]). Gramaglia *et al *could recently show that low NO bioavailability contributes to the genesis of murine cerebral malaria and mice treated with exogenous NO did not develop severe malaria [38]. Similar to NO, CNP may exhibit not only vasodilatory but also anti-inflammatory actions in the vasculature. Scotland *et al *demonstrated that CNP modulates leukocyte-endothelial interactions by down-regulating P-selectin expression directly suppressing leukocyte recruitment. In addition, platelet aggregation and platelet-leukocyte interactions were inhibited by a CNP-mediated suppression of P-selectin expression on platelets indicating an antithrombotic role of CNP [17,18]. As upregulation of adhesion molecules, activation and sequestration of platelets, leukocytes, pRBCs and RBCs are important features of SM pathogenesis [2,25,39], the findings of low CNP levels in the SM group could be interpreted as an insufficient counterregulatory CNP upregulation – with its possible protective anti-inflammatory and vasodilatory effects – probably due to endothelial damage as mentioned above. Similarly to these findings of low CNP levels in SM we had already described significantly lower levels of tissue inhibitor of matrix metalloproteinases-2 (TIMP-2) in the same cohort of patients [40].

However, it has to be considered that plasma levels do not truly reflect the microvascular situation at the site of inflammation. Said *et al *described increased ET-1 levels which were more pronounced in mucosal tissue than in plasma in a rat model of stress-induced mucosal haemorrhage [41]. ET-1 and CNP may act in a paracrine fashion, therefore a correlation between circulating plasma ET-1 and CNP concentration and disease severity might not directly reflect the pathogenetic importance of ET-1 and CNP in severe malaria.

A possible interaction of treatment with plasma levels of ET-1 may also be taken into consideration. An inhibitory effect on ET-1 production in HUVECs has been shown for substances such as ursodeoxycholic acid [42]. Del Pozo *et al *described a downward shift of contractions of isolated thoracic aortic rings induced by endothelin-1 in response to a pretreatment with high concentrations of quinine [43]. All the studied patients with severe malaria were treated with intravenous quinine, according to local guidelines. Whether schizonticidal therapy, such as quinine, and host-derived endothelial peptides, such as ET-1 and CNP, may interact at a circulating enzyme level or could be more pronounced at receptor levels is still insufficiently investigated and, therefore, poses an important research challenge.

## Conclusion

The pathophysiological significance of ET-1 and CNP in malaria is still an open-ended question. Many factors contribute to the course of malaria (eg, pro-inflammatory mediators, sequestration, endothelial activation), thus, it is difficult to assess the contributory role of two specific factors. However, the results of this study provide evidence for an involvement of substances produced in the endothelium in SM pathogenesis and underline the importance of further investigation of the vascular compartment, in particular endothelial cells, and their contribution to the development of the devastating multi-organ complications defining severe malaria.

## Competing interests

The authors declare that they have no competing interests.

## Authors' contributions

AD, PL, RH and ES study design, analysis of data, draft of manuscript; AD and KS sample collection and immunoassays, SI and BL sample collection, critical reading of the manuscript, MR critical reading of the manuscript, PGK: study design, critical reading of the manuscript. All authors read and approved the final manuscript.

## References

[B1] World Health Organization (WHO) World malaria report 2005. http://www.rollbackmalaria.org/wmr2005/.

[B2] Coltel N, Combes V, Hunt NH, Grau GE (2004). Cerebral malaria – a neurovascular pathology with many riddles still to be solved. Curr Neurovasc Res.

[B3] Medana IM, Turner GD (2006). Human cerebral malaria and the blood-brain barrier. Int J Parasitol.

[B4] Wassmer SC, Combes V, Candal FJ, Juhan-Vague I, Grau GE (2006). Platelets potentiate brain endothelial alterations induced by Plasmodium falciparum. Infect Immun.

[B5] Pober JS, Sessa WC (2007). Evolving functions of endothelial cells in inflammation. Nat Rev Immunol.

[B6] Kedzierski RM, Yanagisawa M (2001). Endothelin system: the double-edged sword in health and disease. Annu Rev Pharmacol Toxicol.

[B7] McCarron RM, Wang L, Stanimirovic DB, Spatz M (1993). Endothelin induction of adhesion molecule expression on human brain microvascular endothelial cells. Neurosci Lett.

[B8] Speciale L, Roda K, Saresella M, Taramelli D, Ferrante P (1998). Different endothelins stimulate cytokine production by peritoneal macrophages and microglial cell line. Immunology.

[B9] Wanecek M, Weitzberg E, Rudehill A, Oldner A (2000). The endothelin system in septic and endotoxin shock. Eur J Pharmacol.

[B10] Piechota M, Banach M, Irzmanski R, Barylski M, Piechota-Urbanska M, Kowalski J, Pawlicki L (2007). Plasma endothelin-1 levels in septic patients. J Intensive Care Med.

[B11] Petkova SB, Huang H, Factor SM, Pestell RG, Bouzahzah B, Jelicks LA, Weiss LM, Douglas SA, Wittner M, Tanowitz HB (2001). The role of endothelin in the pathogenesis of Chagas' disease. Int J Parasitol.

[B12] Schuetz P, Stolz D, Mueller B, Morgenthaler NG, Struck J, Mueller C, Bingisser R, Tamm M, Christ-Crain M (2008). Endothelin-1 precursor peptides correlate with severity of disease and outcome in patients with community acquired pneumonia. BMC Infect Dis.

[B13] Wenisch C, Wenisch H, Wilairatana P, Looareesuwan S, Vannaphan S, Wagner O, Graninger W, Schonthal E, Rumpold H (1996). Big endothelin in patients with complicated *Plasmodium falciparum *malaria. J Infect Dis.

[B14] Machado FS, Desruisseaux MS, Nagajyothi, Kennan RP, Hetherington HP, Wittner M, Weiss LM, Lee SC, Scherer PE, Tsuji M, Tanowitz HB (2006). Endothelin in a murine model of cerebral malaria. Exp Biol Med (Maywood).

[B15] Basilico N, Mondani M, Parapini S, Speciale L, Ferrante P, Taramelli D (2004). *Plasmodium falciparum *parasitized red blood cells modulate the production of endothelin-1 by human endothelial cells. Minerva Med.

[B16] Fowkes RC, McArdle CA (2000). C-type natriuretic peptide: an important neuroendocrine regulator?. Trends Endocrinol Metab.

[B17] Scotland RS, Ahluwalia A, Hobbs AJ (2005). C-type natriuretic peptide in vascular physiology and disease. Pharmacol Ther.

[B18] Scotland RS, Cohen M, Foster P, Lovell M, Mathur A, Ahluwalia A, Hobbs AJ (2005). C-type natriuretic peptide inhibits leukocyte recruitment and platelet-leukocyte interactions via suppression of P-selectin expression. Proc Natl Acad Sci USA.

[B19] Sylla EH, Kun JF, Kremsner PG (2000). Mosquito distribution and entomological inoculation rates in three malaria-endemic areas in Gabon. Trans R Soc Trop Med Hyg.

[B20] Wildling E, Winkler S, Kremsner PG, Brandts C, Jenne L, Wernsdorfer WH (1995). Malaria epidemiology in the province of Moyen Ogoov, Gabon. Trop Med Parasitol.

[B21] World Health Organization (WHO) (2000). Severe falciparum malaria. Trans R Soc Trop Med Hyg.

[B22] Helbok R, Dent W, Nacher M, Lackner P, Treeprasertsuk S, Krudsood S, Wilairatana P, Silachamroon U, Looareesuwan S, Schmutzhard E (2005). The use of the multi-organ-dysfunction score to discriminate different levels of severity in severe and complicated *Plasmodium falciparum *malaria. Am J Trop Med Hyg.

[B23] Helbok R, Issifou S, Matsiegui PB, Lackner P, Missinou MA, Kombila D, Dent W, Schmutzhard E, Kremsner PG (2006). Simplified multi-organ dysfunction score predicts disability in African children with *Plasmodium falciparum *malaria. Am J Trop Med Hyg.

[B24] Planche T, Krishna S, Kombila M, Engel K, Faucher JF, Ngou-Milama E, Kremsner PG (2001). Comparison of methods for the rapid laboratory assessment of children with malaria. Am J Trop Med Hyg.

[B25] Hunt NH, Grau GE (2003). Cytokines: accelerators and brakes in the pathogenesis of cerebral malaria. Trends Immunol.

[B26] Rogerson SJ, Grau GE, Hunt NH (2004). The microcirculation in severe malaria. Microcirculation.

[B27] Suga S, Nakao K, Itoh H, Komatsu Y, Ogawa Y, Hama N, Imura H (1992). Endothelial production of C-type natriuretic peptide and its marked augmentation by transforming growth factor-beta. Possible existence of "vascular natriuretic peptide system". J Clin Invest.

[B28] Blais V, Fugere M, Denault JB, Klarskov K, Day R, Leduc R (2002). Processing of proendothelin-1 by members of the subtilisin-like pro-protein convertase family. FEBS Lett.

[B29] Wu C, Wu F, Pan J, Morser J, Wu Q (2003). Furin-mediated processing of Pro-C-type natriuretic peptide. J Biol Chem.

[B30] Basilico N, Speciale L, Parapini S, Ferrante P, Taramelli D (2002). Endothelin-1 production by a microvascular endothelial cell line treated with *Plasmodium falciparum *parasitized red blood cells. Clin Sci (Lond).

[B31] Hemmer CJ, Lehr HA, Westphal K, Unverricht M, Kratzius M, Reisinger EC (2005). *Plasmodium falciparum *malaria: reduction of endothelial cell apoptosis in vitro. Infect Immun.

[B32] Lackner P, Burger C, Pfaller K, Heussler V, Helbok R, Morandell M, Broessner G, Tannich E, Schmutzhard E, Beer R (2007). Apoptosis in experimental cerebral malaria: spatial profile of cleaved caspase-3 and ultrastructural alterations in different disease stages. Neuropathol Appl Neurobiol.

[B33] Ramzy D, Rao V, Tumiati LC, Xu N, Sheshgiri R, Miriuka S, Delgado DH, Ross HJ (2006). Elevated endothelin-1 levels impair nitric oxide homeostasis through a PKC-dependent pathway. Circulation.

[B34] Tedgui A, Mallat Z (2001). Anti-inflammatory mechanisms in the vascular wall. Circ Res.

[B35] Kremsner PG, Winkler S, Wildling E, Prada J, Bienzle U, Graninger W, Nussler AK (1996). High plasma levels of nitrogen oxides are associated with severe disease and correlate with rapid parasitological and clinical cure in *Plasmodium falciparum *malaria. Trans R Soc Trop Med Hyg.

[B36] Kun JF, Mordmuller B, Lell B, Lehman LG, Luckner D, Kremsner PG (1998). Polymorphism in promoter region of inducible nitric oxide synthase gene and protection against malaria. Lancet.

[B37] Kun JF, Mordmuller B, Perkins DJ, May J, Mercereau-Puijalon O, Alpers M, Weinberg JB, Kremsner PG (2001). Nitric oxide synthase 2 (Lambarene) (G-954C), increased nitric oxide production, and protection against malaria. J Infect Dis.

[B38] Gramaglia I, Sobolewski P, Meays D, Contreras R, Nolan JP, Frangos JA, Intaglietta M, Heyde HC van der (2006). Low nitric oxide bioavailability contributes to the genesis of experimental cerebral malaria. Nat Med.

[B39] Miller LH, Baruch DI, Marsh K, Doumbo OK (2002). The pathogenic basis of malaria. Nature.

[B40] Dietmann A, Helbok R, Lackner P, Issifou S, Lell B, Matsiegui PB, Reindl M, Schmutzhard E, Kremsner PG (2008). Matrix metalloproteinases and their tissue inhibitors (TIMPs) in *Plasmodium falciparum *malaria: serum levels of TIMP-1 are associated with disease severity. J Infect Dis.

[B41] Said SA, El Mowafy AM (1998). Role of endogenous endothelin-1 in stress-induced gastric mucosal damage and acid secretion in rats. Regul Pept.

[B42] Ma J, Iida H, Jo T, Takano H, Oonuma H, Morita T, Toyo-Oka T, Omata M, Nagai R, Okuda Y, Yamada N, Nakajima T (2004). Ursodeoxycholic acid inhibits endothelin-1 production in human vascular endothelial cells. Eur J Pharmacol.

[B43] del Pozo BF, Perez-Vizcaino F, Villamor E, Zaragoza F, Tamargo J (1996). Stereoselective effects of the enantiomers, quinidine and quinine, on depolarization- and agonist-mediated responses in rat isolated aorta. Br J Pharmacol.

